# Functional results of multiple revision anterior cruciate ligament with anterolateral tibial tunnel associated with anterolateral ligament reconstruction

**DOI:** 10.1186/s43019-022-00153-3

**Published:** 2022-05-08

**Authors:** Camilo Partezani Helito, Andre Giardino Moreira da Silva, Tales Mollica Guimarães, Marcel Faraco Sobrado, José Ricardo Pécora, Gilberto Luis Camanho

**Affiliations:** 1 Grupo de Joelho, Instituto de Ortopedia e Traumatologia, Hospital das Clínicas HCFMUSP, Faculdade de Medicina, Universidade de São Paulo, Rua Dr. Ovídio Pires de Campos, 333 - Cerqueira Cesar, São Paulo, SP CEP: 05403-010 Brazil; 2 Hospital Sírio Libanês, Rua Dona Adma Jafet, 91 - Bela Vista, São Paulo, SP CEP 01308-050 Brazil

**Keywords:** Revision ACL reconstruction, Enlarged ACL tibial tunnel, Anterolateral ligament, ACL, ALL

## Abstract

**Background:**

Revision anterior cruciate ligament (ACL) reconstructions are usually complex owing to previous tunnels. The objective of this study is to report the results of a revision ACL reconstruction technique with a tibial tunnel performed from the anterolateral plateau associated with an anterolateral ligament (ALL) reconstruction.

**Methods:**

Patients with at least two ACL reconstructions that failed and who had significant enlargement and confluence of tunnels in the medial tibial plateau and underwent revision ACL reconstruction associated with ALL reconstruction with the tibial tunnel for the ACL performed from the lateral plateau between 2017 and 2019 were evaluated. All patients were evaluated by physical examination, International Knee Documentation Committee (IKDC), and Lysholm functional scales.

**Results:**

Six patients who underwent this surgical procedure were evaluated. All patients were sports practitioners and presented a grade 3 pivot shift. The mean age was 28.5 ± 8.2 years, and the mean follow-up time was 34.1 ± 12.8 months. No patient had a new graft rupture, but three (50%) had grade 1 pivot shift. Four patients had minor complications with no clinical impact on the final result. All except one patient were able to return to pre-injury type of sports, at a mean time of 14.6 ± 2.3 months after surgery.

**Conclusion:**

The anterolateral tibial tunnel technique using an Achilles tendon allograft for revision ACL reconstruction after multiple failures associated with an ALL reconstruction showed good results and no major complications. The anterolateral tunnel can be considered a good alternative in cases of medial tibial confluence or significant enlargement of the medial tunnels in re-revision procedures.

## Background

Anterior cruciate ligament (ACL) injuries are quite frequent in sports practice. In the young population, the most accepted treatment is reconstruction, but studies show that conservative treatment and eventual repair are also possible, with acceptable results [1]. Although most cases evolve with good results, some patients present graft rupture. The failure rate is around 7% in the general population, but it can reach 20% in specific groups [2, 3].

Revision ACL reconstructions are usually more complex owing to previous tunnels, fixation materials, and a significant number of associated injuries [4]. When there are enlarged tunnels, the graft choice for revision must often be meticulous, and the reason for the enlargement must be understood [5]. Tunnels with a diameter of more than 16 mm should not be used, and in these situations, it is preferable to change the tunnel or to perform a two-stage revision, filling the tunnels with bone grafting in the first stage and doing the ligament reconstruction in the second stage. A systematic review by Colatruglio et al. [6] found no differences between the one-stage and two-stage revision, although the authors conclude that the evidence is retrospective and limited.

For the femoral tunnels, changing the drilling direction normally is not a major issue. The construction with the transportal, transtibial, and, mainly, outside-in techniques allows the new tunnels to be performed in directions different from the old ones. Pioger et al. [7] evaluated 409 revisions performed in a single stage. The femoral tunnel was drilled with the outside-in technique, with no major technical problems and no need to perform the procedure in two stages. Although it is possible to change the angle in the tibial tunnel drilling, often there is not much space for drilling in the medial tibial plateau, especially when at least two tunnels have already been performed. Thus, an option not commonly used and studied in literature is the perforation of the new tunnel from the lateral plateau, avoiding the previous tunnels and fixation materials in the medial tibial plateau [8]. In cases of revision, many authors also recommend the associated extra-articular augmentation procedure, which also increases the number of tunnels and fixations, making the procedure more difficult [9, 10].

Thus, the objective of this study is to report a revision ACL technique with a tibial tunnel performed from the anterolateral plateau associated with an anterolateral ligament (ALL) reconstruction, in addition to describing its clinical results with a minimum of 2 years of follow-up. We hypothesize that there will be no technical difficulties in performing the technique, and the functional results will be adequate. Furthermore, the benefit of this technique is to enable the revision ACL reconstructions after multiple failures in a single stage, also minimizing the use of grafts, tunnels, and fixations.

## Methods

Patients with at least two ACL reconstructions that failed and who had significant enlargement and confluence of tunnels in the medial tibial plateau and underwent revision ACL reconstruction associated with ALL reconstruction with the tibial tunnel for the ACL performed from the lateral plateau between 2017 and 2019 were included. In the study period, 14 re-revision surgeries were performed, but this procedure was indicated only if a performing a medial tibial tunnel was not possible and when a single-stage revision was planned. Patients in whom surgery with tibial tunnel through the medial plateau was possible submitted to reconstructions associated with posterior cruciate ligament (PCL), medial collateral ligament (MCL), and posterolateral corner (PLC), or associated osteotomies for axis or slope correction were not included.

**Fig. 1:**
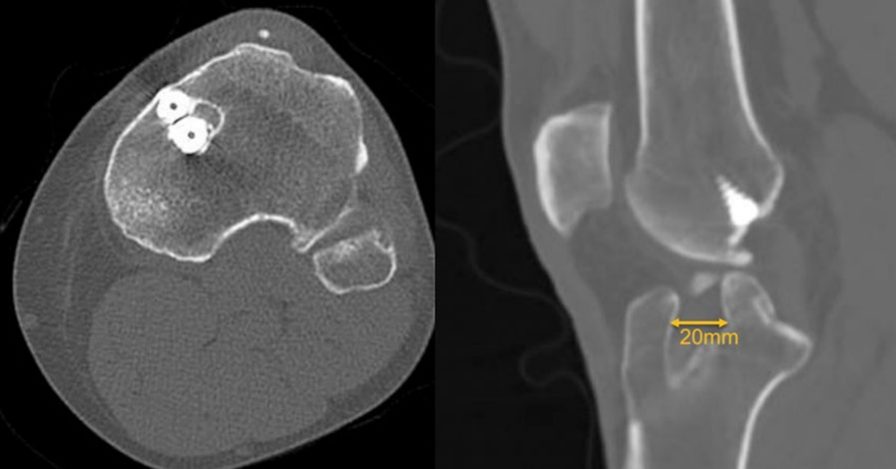
Tomography image in the axial and coronal planes showing a patient with an anterior cruciate ligament (ACL) injury and confluence of tunnels in the medial plateau. The largest diameter of the tunnel in the coronal plane was 20 mm

All patients were evaluated preoperatively with knee weight-bearing radiographs, long-leg radiographs to assess the limb axis, lateral tibial radiograph to measure the slope, tomography to assess the positioning and confluence of previous tunnels, and magnetic resonance imaging, in addition to a careful physical examination for ligament instabilities of the knee, which was repeated after anesthesia to confirm associated injuries (Fig. 1).

ACL surgery with the lateral tibial tunnel was planned and indicated when there were two or more previous tunnels in the medial plateau and when the construction of a third tunnel could make an adequate reconstruction impossible. It was considered when there was already confluence between the previous tunnels, when the bone bridge between the tunnels was smaller than 3 mm and a new perforation could weaken the walls and generate confluence, and when the perforation in the right position would connect the previous tunnels. Thus, once the lateral tunnel was planned, the medial part of the plateau was not violated during surgery. In all situations other than those previously mentioned, this technique was not indicated and a conventional medial tibial tunnel reconstruction was performed. Relative contra-indications of this procedure include patients with previous fixations materials and lateral tunnels that could impair the new tunnel for the revision ACL reconstruction.

### Surgical procedure

#### Patient preparation

Patients were placed in horizontal dorsal decubitus on a radiolucent table so fluoroscopy could be used if necessary. Patients were operated under spinal anesthesia, with a tourniquet placed at the root of the thigh.

#### Graft preparation

A long Achilles tendon from a tissue bank was prepared with a bone plug. The tendon was prepared with an 11–12 mm bone plug, soft tissue for ACL reconstruction with 10–11 mm, and soft tissue for ALL reconstruction with 7 mm. The tip of the graft used for the ALL was prepared with high-strength sutures.

#### Femoral tunnel for ACL reconstruction

The femoral tunnel for combined ACL and ALL reconstruction was performed using the outside-in technique. Tunnel entry occurred posterior and proximal to the lateral epicondyle, and the tunnel exit in the articular region of the ACL femoral footprint, closer to the anteromedial bundle. After drilling, the tunnel was cleaned, removing any remaining grafts and screws from previous surgeries. It is important to leave the tunnel walls with bleeding bone for better graft integration. In the case of very sclerotic walls, “microfracture” type perforations in the inner part of the tunnel wall can be performed.

#### Lateral tibial tunnel for ACL reconstruction

**Fig. 2:**
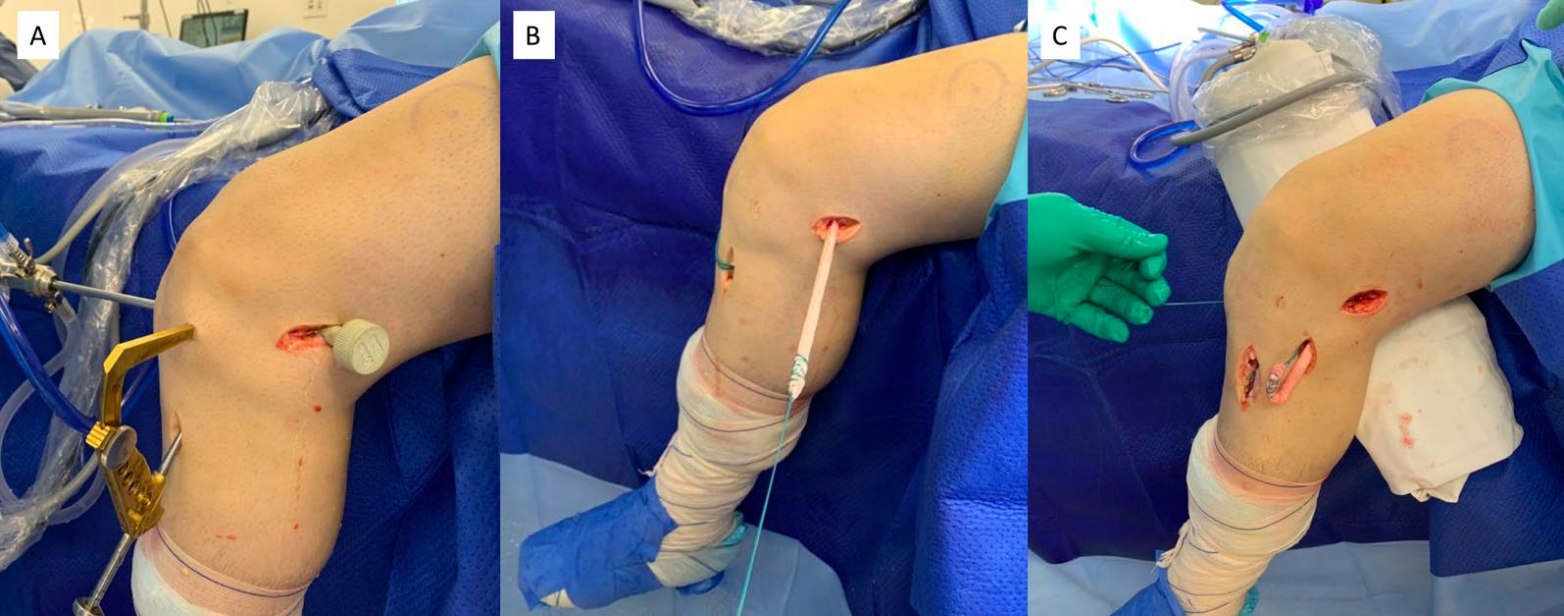
Image of the revision anterior cruciate ligament (ACL) reconstruction with a tibial tunnel from the lateral plateau. The tip of the tibial tunnel guide enters through the anterolateral portal, and the guidewire enters the tibia close to the tibial crest. In the image, it is possible to see that the combined femoral tunnel for the ACL and the anterolateral ligament has already been performed (**A**). The Achilles tendon allograft is passed from the tibia to the femur (**B**), and the remaining portion of the graft is passed below the iliotibial band toward the anterolateral tibia for the anterolateral ligament reconstruction (**C**)

To perform the tibial tunnel through the lateral plateau, an access immediately lateral to the tibial crest is performed. The direction of the tunnel must be as vertical as possible to avoid sharper angulation from the graft in the joint. To perform the tunnel through the lateral access, it is important to detach the muscles from the anterolateral region of the tibia to access the local bone. The tibial guide with 60° of angulation is then positioned in the center of the ACL footprint, entering the anterolateral portal (Fig. 2). The passage of the guidewire must be performed calmly to prevent it from slipping into the proximal tibiofibular joint since the tibia is straighter in this region compared with the medial plateau; then the exit of the guidewire from the joint is checked with the arthroscopy camera. After drilling the tunnel, the camera must be placed inside the tunnel to check the integrity of the walls. Next, a bone shaver must be placed inside the tunnel to flatten the posterolateral region of the tunnel entrance at the joint. This step is important for the graft curvature to be as smooth as possible.

#### Tibial tunnel for ALL reconstruction

The tibial tunnel for ALL reconstruction should be performed between Gerdy’s tubercle and the fibular head, about 5–10 mm below the lateral tibial plateau. The tunnel must be performed from lateral to medial, exiting posteriorly to the current anterolateral tunnel and the previous anteromedial tunnels. In case of doubt, fluoroscopy can be used, although it has not been necessary to date in our clinical practice. After making the tunnel, the camera must be placed inside it to check the integrity of the walls.

#### Graft passage and fixation

**Fig. 3:**
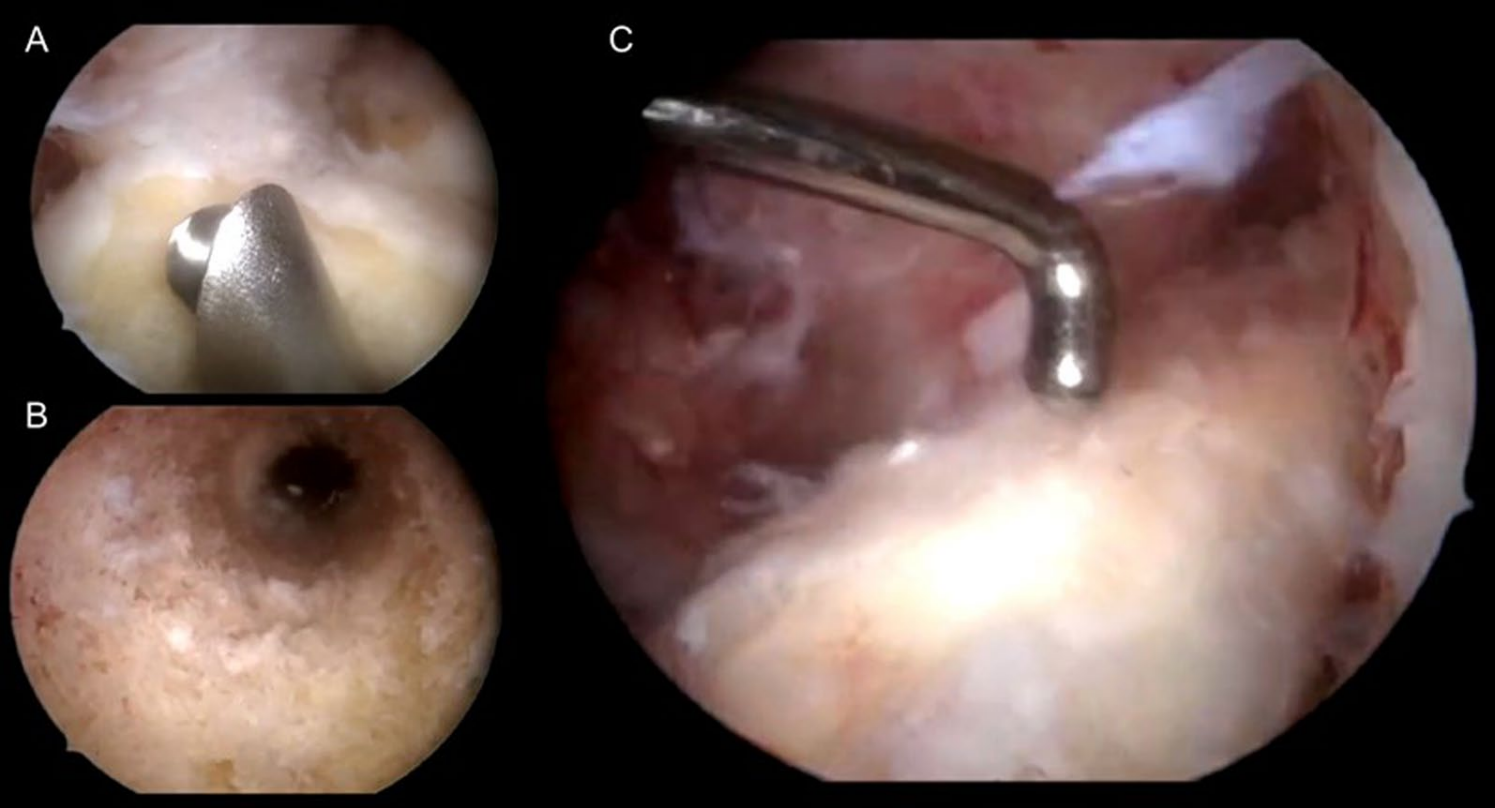
Intraoperative images showing the use of a bone shaver blade to smooth the graft curvature at the entrance to the tibia (**A**), the tibial tunnel with intact walls and without confluence with previous medial tunnels (**B**), and the appearance of the Achilles tendon allograft after fixation (**C**)

**Fig. 4:**
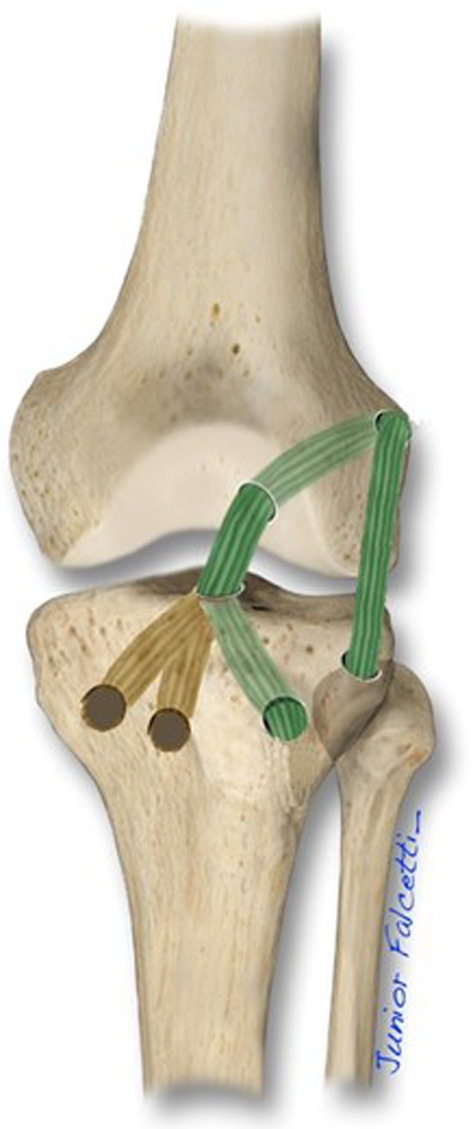
Schematic drawing of the combined anterior cruciate ligament (ACL) reconstruction and the anterolateral ligament with the lateral tibial tunnel. The previous medial tunnels are shown in brown

After performing the tunnels, the Achilles tendon allograft is passed from the tibia to the femur, leaving the bone plug in the tibial tunnel and the part for ALL reconstruction outside the femoral tunnel. Initially, femoral fixation is performed in the combined femoral tunnel with an interference screw from the outside-in. At the time of fixation, it is important to keep the entire bone plug inside the tibial tunnel. After femoral fixation, the tibial tunnel of the ACL is also fixed using an interference screw and in 30° flexion of the knee. Finally, fixation of the ALL is performed. The remaining Achilles graft is passed under the iliotibial tract and inserted into the tibia. Fixation is performed with an absorbable interference screw from lateral to medial with full extension and neutral knee rotation (Figs. 3, 4).

#### Postoperative care and rehabilitation

All patients were allowed partial weight-bearing as tolerated from the initial postoperative period. Progression to full weight-bearing was allowed when patients had good quadriceps control. Gaining range of motion was also authorized from the first postoperative period, without using any knee immobilizer. The return to contact sports was authorized at least 12 months after the operation, as long as the patient was free of pain, without effusion, with a complete range of motion and good muscular control.

All patients were evaluated by physical examination, including KT-1000 and pivot shift. Patients were evaluated using the International Knee Documentation Committee (IKDC) and Lysholm functional scales at the last postoperative visit. All complications have been documented.

## Results

Six patients who underwent a second revision ACL associated with ALL reconstruction with lateral tibial tunnel and a long Achilles allograft were evaluated. All patients were recreational sports practitioners and presented a grade 3 pivot shift preoperatively. The mean age of patients was 28.5 ± 8.2 years, and the mean follow-up time was 34.1 ± 12.8 months. According to tomographic evaluation, three patients presented confluence between the previous tunnels, two patients presented a bone bridge between the previous tunnels smaller than 3 mm, and in one patient the perforation in the correct position would connect the previous tunnels. The preoperative data are presented in Table 1.

**Table 1:** Demographic and preoperative data of patients included in the study

Age (years)	28.5 ± 8.2
Gender	Male 3 (50%)
	Female 3 (50%)
Patients with hyperlaxity	3 (50%)
Posterior tibial slope (degrees)	9.3 ± 2.7 (range 5–12)
Tunnel diameter (mm)	16.8 ± 3.3 (range 13–21) _
Time from injury to primary reconstruction (months)	2 ± 0.9
Time from second injury to first revision reconstruction (months)	4.6 ± 1.3
Time from third injury to second revision reconstruction (months)	15.6 ± 8.6
Graft used in first reconstruction	Patellar 1 (16.7%) Hamstrings 5 (83.3%)
Graft used in second reconstruction	Patellar 4 (66.6%)
	Hamstrings 1 (16.7%)
	Contralateral hamstrings 1 (16.7%)
Preoperative KT-1000 (mm)	9.3 ± 0.5
Preoperative pivot shift	100% grade 3
Previous partial medial meniscectomy	4 (66.7%)
Previous partial lateral meniscectomy	1 (16.7%)
Sports	Soccer 3 (50%)
	Handball 2 (33.3%)
	Triathlon 1 (16.7%)

For tunnel diameter measurement, the larger tunnel diameter in any plane was considered. When there were two separated tunnels, the one with larger diameter was considered

No patient had a new graft rupture in the postoperative period, but three (50%) had grade 1 pivot shift. Four patients had minor complications with no clinical impact on the final result, two superficial infections treated with oral antibiotics, one cyclops lesion treated with arthroscopy debridement, and one flexion loss of 10°, where the patient accepted the result and no further intervention was performed. All except one patient were able to return to pre-injury type of sports, at a mean time of 14.6 ± 2.3 months after surgery. However, only two patients were able to return to the pre-injury level. Postoperative patient data are described in Table 2.

**Table 2:** Postoperative data of patients included in the study

Follow-up time (months)	34.1 ± 12.8
Intra-articular ACL graft diameter (mm)	10.7 ± 0.5
Extra-articular ALL graft diameter (mm)	7 ± 0
Length of lateral tibial tunnel (mm)	43.7 ± 5.7
Postoperative KT-1000	2.0 ± 1.1
Postoperative pivot shift	Grade 0 3 (50%)
	Grade 1 3 (50%)
Subjective IKDC	79.1 ± 6.3
Lysholm	82.8 ± 5.1
Lateral pain	100%
Lateral pain time (months)	3.8 ± 2.1
Current meniscal injury	4 (66.7%)
Injured meniscus	Medial 2 (50%)
	Lateral 2 (50%)
Treatment of meniscal injury	Suture 1 (25%)
	Meniscectomy 3 (75%)
Complications	Superficial infection 2 (33.3%)
	Cyclops 1 (16.7%)
	10° flexion loss 1 (16.7%)
Return to sport	5 (83.3%)
Return time to sport (months)	14.6 ± 2.3
Return to pre-injury level	2 (33.3%)

## Discussion

The main finding in this study is the possibility of performing, with good results, multiple ACL revisions in a single-stage procedure, associated with extra-articular reconstruction and with a single graft through an anterolateral access to perform the tibial tunnel. Furthermore, the clinical results of these highly complex cases were satisfactory, and complications did not interfere with the results.

Indications for the two-stage revision reconstruction are situations in which it is impossible to place the new tunnel in a good position, generating confluence with previous tunnels, or when there is a tunnel enlargement, usually considered to be greater than 16 mm [11]. Richter et al. [12] consider tunnels from 14 to 15 mm as an indication for a two-stage revision. In these situations, bone grafting of the tunnels with subsequent revision after the consolidation is recommended, usually 3–4 months after the single-stage procedure [12]. Thomas et al. [13] performed the two-stage reconstruction with a mean of 5.8 months after bone grafting. Despite providing a safer revision in terms of tunnel placement, the patient takes longer to return to their sports activity in the two-stage revision, which is related to more degenerative changes and chondral and meniscal injuries [13]. Kim et al. [14] also concluded that two-stage revision has worse outcomes, especially in more active patients. Thomas et al. [13] found a mean IKDC of only 61.2 after two-stage revision.

The confluence of tunnels and the need for grafting have been more studied for femoral bone defects, with less literature on tibial tunnels. Despite enlarged tunnels, the study by Pioger et al. [7] evaluated 409 patients undergoing single-stage revision and found no postoperative differences between patients who had enlarged tunnels and those who did not. The authors conclude that using the outside-in technique for the femur allows an adequate revision without the need for bone grafting.

Few studies have focused on finding solutions other than grafting for enlarged tibial tunnels. A biomechanical study by Van der Bracht et al. [8] concluded that it is possible to use an anterolateral tunnel in the tibia with adequate knee stability to avoid an eventual medial defect, and a clinical study by Keyhani et al. [15] with 25 patients also showed promising results in the case of the first ACL revision. Some authors have studied the benefit of performing reconstruction with a lateral tibial tunnel for PCL reconstruction for reasons other than enlarged tunnels [14, 16]. Other techniques described include using screws with a much larger diameter to fill the tunnel gaps [17] or using an impaction bone graft, as described by Demyttenaere et al. in the evaluation of eight patients. [18].

Recently, anterolateral reconstructions have been increasingly indicated in conjunction with ACL revisions [2, 19, 20]. Two recent consensuses include the revision as a possible indication for lateral reinforcement, and studies focused on revision ACL reconstruction showed the benefits of an associated extra-articular procedure [21–23]. The addition of an ALL reconstruction associated with an ACL revision adds even more difficulty in case of enlarged tunnels and the need for grafts, especially in cases of multiple revisions. Fernandez et al. [24] described a long Achilles tendon technique for revision ACL reconstruction and ALL reconstruction, but the authors performed the procedure in two stages and with the medial tibial tunnel, precisely to fill the bone defects initially, different from our proposal for single-stage reconstruction with an anterolateral tibial tunnel. Slope correction osteotomies also can be indicated in a re-revision ACL reconstruction, but this procedure is normally indicated in patients with at least 13° of tibial slope. As none of the patients in this series presented this value, no slope osteotomy was performed.

The technique described in this paper can solve the problems of confluence of tibial tunnels through the anterolateral access. Even with an eventual partial confluence in the joint portion, the average tunnel length of 43.7 mm allows sufficient wall for adequate fixation with interference screws, as described by Van der Bracht et al. [25]. The only major care to be taken is the flattening of the tunnel joint exit to avoid sharper angulation of the graft curvature. Care should also be taken to avoid coalition between the anterolateral ACL tibial tunnel and the ALL tibial tunnel, but in this series the anterolateral tunnel did not interfere with the ALL tunnel either. Care should be taken with the direction of the ALL tunnel posterior to the ACL tunnel, but the tibia space is usually sufficient. Fluoroscopy can be used if there is any doubt regarding possible confluence. As demonstrated by Pioger et al. [7], we did not find a problem with the construction of the femoral tunnel using the outside-in technique. A single tunnel for the ACL and ALL also minimizes complications by decreasing the number of perforations [26].

Regarding clinical results, the technique presented was compatible with the literature. As for primary reconstructions, revisions already tend to have worse functional results and a lower rate of return to sport, and multiple revisions tend to have even worse results [27]. Yoon et al. [28] evaluated 20 patients submitted to re-revision ACL reconstruction and found a 30% failure rate, in addition to a mean IKDC of 60.5. In this study, the authors concluded that the cases of re-revision evolve worse than those of revision. Colatruglio et al. [6] did a systematic review of 13 studies and 524 patients and found an IKDC of 66.6 for a single-stage revision and 65.9 for a two-stage revision. Our study found IKDC and Lysholm results inferior to the results of a primary reconstruction but still satisfactory considering the revision context. According to the patient acceptable symptom state (PASS) for IKDC, only one patient did not reach the minimum score of 75.9 [29]. Patients submitted to primary ACL and ALL reconstruction in a recent study had mean IKDC and Lysholm values around 90 [30].

The retrospective nature of the sample and the small number of patients are some of the limitations of this study. In addition, we lack a control group in which the two-stage revision could have been performed, initially filling the medial tunnels with bone graft. However, the objective of the study was to present the initial functional results, safety, reproducibility, and possible complications of single-stage procedure with the anterolateral tibial tunnel, which justifies the absence of the control group in this study.

## Conclusion

The anterolateral tibial tunnel technique using an Achilles tendon allograft for revision ACL reconstruction after multiple failures associated with an ALL reconstruction showed good results and no major complications. The anterolateral tunnel can be considered a good alternative in cases of medial tibial confluence or significant enlargement of the medial tunnels in re-revision procedures.

## Data Availability

All data generated or analyzed during this study are included in this published article.

## Abbreviations

IKDC: International Knee Documentation Committee
ACL: Anterior cruciate ligament
ALL: Anterolateral ligament
PCL: Posterior cruciate ligament
MCL: Medial collateral ligament
PLC: Posterolateral corner
